# Computational simulations of coronary artery bifurcation stenting using realistic plaque distribution and materials

**DOI:** 10.1038/s41598-025-16258-0

**Published:** 2025-10-01

**Authors:** Wei Wu, Shijia Zhao, Rakshita Ramesh Bhat, Yash Vardhan Trivedi, Parth Munjal, Rahul Chikatimalla, Ruben K.A. Tapia-Orihuela, Hammad Zafar, Haritha Darapaneni, Komal Arora, Changkye Lee, Yiannis S. Chatzizisis

**Affiliations:** Center for Digital Cardiovascular Innovations, Division of Cardiovascular Medicine, Leonard M. Miller School of Medicine, University of Miami Health System, University of Miami, 1120 NW 14th Street, Suite 1124, Miami, FL 33136 USA

**Keywords:** Computational simulations, Finite element analysis, 3D reconstruction, Coronary bifurcation stenting, Plaque materials, Computational models, Interventional cardiology, Atherosclerosis

## Abstract

Coronary bifurcation stenting is a technically demanding procedure that benefits from precise preoperative planning. Finite element analysis (FEA) offers a powerful framework for simulating stent deployment, with full-plaque (FP) models representing the most detailed and physiologically realistic approach. In this study, we developed and validated an FP finite element model for coronary bifurcation stenting, aiming to establish a reference standard for future model assessments. High-resolution pre-stenting optical coherence tomography (OCT) data from nine patient cases were used to reconstruct anatomically accurate 3D models, incorporating detailed plaque compositions. Stent deployment was simulated following real clinical procedural steps. Model accuracy was assessed by comparing minimal lumen diameter (MLD) between simulations and post-stenting OCT measurements using Bland–Altman analysis. The FP model reliably reproduced multi-step stenting procedures, capturing vessel remodeling and stent deformation with high fidelity. The analysis showed a mean bias of 0.07 mm (2.1% error) with 95% limits of agreement from − 0.38 mm to 0.51 mm. These results demonstrate the model’s high accuracy in replicating real-world stenting outcomes. Beyond validation, the FP model offers broad applications. It can serve as a benchmark for evaluating simplified, faster simulation tools, support patient-specific procedural planning, and enable inverse modeling to extract biomechanical properties of plaques. This study establishes the FP model as a robust and versatile platform with potential for future integration into real-time clinical workflows.

**Supplementary Information:**

The online version contains supplementary material available at 10.1038/s41598-025-16258-0.

## Introduction

 Coronary bifurcations are unique anatomical locations within the coronary tree that are highly susceptible to atherosclerosis and restenosis^1,2^. Percutaneous coronary intervention (PCI) in these regions is inherently complex, especially for multi-step bifurcation stenting, as it requires precise planning to ensure optimal stent expansion and vascular integrity^3^. Accurate modeling and simulation of stent deployment are critical for optimizing stenting strategies and improving patient outcomes. Finite element analysis (FEA) has emerged as an essential tool for simulating stent deployment, providing insights into the biomechanical behavior of coronary bifurcations during PCI^4,5^.

Among the various FEA approaches, full-plaque (FP) models—which incorporate detailed representations of the lumen, vessel wall, and plaque components—are regarded as the most accurate means of simulating stent deployment. By capturing the intricate interactions between the stent, vessel wall, and heterogeneous plaque components (fibrosis, fibrolipid, and calcified zones), the FP model provides an unparalleled level of detail^6,7^. Despite this complexity, FP models offer unmatched predictive accuracy, making them the most comprehensive approach for evaluating stent-induced changes in vessel geometry^7^.

This study presents the development, validation, and performance of the FP model using nine patient-specific coronary bifurcation cases. The aim of this study is to validate the FP model’s accuracy against lumen and stent deformation, using post-stenting optical coherence tomography (OCT) as the ground truth. After the validation, the FP models’ clinical and biomechanical implications were discussed. Compared to previous models, the novelty of our FP model lies in its integration of high-resolution OCT imaging to precisely reconstruct detailed plaque compositions and anatomically accurate vessel geometries, combined with rigorous validation against post-stenting OCT outcomes.

## Materials and methods

### Study population

All methods were performed following the relevant guidelines and regulations. The study was approved by the ethics committee of Teikyo University (IRB approval number #15-159-2), and written informed consent was obtained from all subjects. Nine patients with diseased coronary artery bifurcations who underwent PCI with stenting were selected for this study. Basic stenting information is summarized in Table 1, where two types of stents were used (Resolute Onyx and Resolute Integrity).

**Table 1:** PCI procedure.

Case	Main Vessel	Side Branch	Steps
1	LAD	D3	- Predilatation in the MV with semi-compliant Glider 2.0 mm x 4 mm at 11 atm pressure - MV stenting with Resolute Onyx stent 2.5 mm x 18 mm at 14 atm pressure - POT in the MV with non-compliant balloon Hiryu Plus 3.0 mm x 6 mm at 10 atm pressure - Post-dilatation in the MV 4 times - SB dilatation with non-compliant balloon Hiryu Plus 2.5 mm x 6 mm at 6 atm pressure
2	LAD	D1	- Predilatation 3 times consecutive using non-compliant Euphora balloon 3.0 mm x 12 mm at 16 atm pressure - MV stenting using Resolute Integrity stent 3.0 mm x 38 mm at 12 atm pressure - SB strut opening with semi-complinat balloon Ikazuchi 2.5 mm x 15 mm at 6 atm pressure - KBI with non-compliant Euphora balloon 3.0 mm x 12 mm at 6 atm pressure in the MV and semi-compliant Ikazuchi balloon 2.0 mm x 15 mm at 6 atm pressure in the SB - POT with non-compliant Euphora balloon 3.0 mm x 12 mm at x pressure
3	LAD	D1	- MV stenting using Resolute Integrity stent 3.5 mm x 18 mm at 8 atm pressure - Post-dilatation in the MV using non-compliant TREK balloon 3.75 mm x 18 mm at 8 atm pressure - Post-dilatation in the SB using semi-compliant Glider balloon 2.5 mm x 4 mm at 6 atm pressure-
4	LAD	D1	- Predilatation in the MV 4 times with non-compliant Euphora balloon 2.5 mm x 15 mm at 14 atm pressure - MV stenting using Resolute Integrity stent 2.5 mm x 18 mm at 12 atm pressure KBI with non-compliant Euphora balloon 2.5 mm x 15 mm at 14 atm pressure in the MV and non-compliant Euphora balloon 2.0 mm x 15 mm at 8 atm pressure in the SB
5	LAD	D1	- MV stenting with Resolute Onyx stent 2.5 mm x 18 mm at 18 atm pressure - POT in the MV 2 times with non-compliant Pantera LEO balloon 3.5 mm x 8 mm at 18 atm pressure - POT in the SB with non-compliant Hiryu Plus balloon 2.25 mm x 12 mm at 14 atm pressure
6	LAD	D2	- Predilatation in the MV with non-compliant balloon 2.5 mm x 12 mm at x pressure - MV stenting with Resolute Onyx stent 3.5 mm x 26 mm at 8 atm pressure - Post dilatation in the MV 3 times - KBI with non-compliant balloon Hiryu Plus 3.0 mm x 12 mm at 22 atm in the MV and non-compliant balloon Sapphire Upro 2.0 mm x 12 mm at 10 atm pressure in the SB
7	LAD	D3	- Predilatation in the MV with semi-compliant Euphora 2.25 mm x 12 mm at 6 atm pressure - MV stenting with Resolute Onyx 3.0 mm x 34 mm at 7 atm pressure - Post-dilatation 8 times in the MV with non-compliant Euphora balloon 3.0 mm x 8 mm at 15 atm pressure - Post-dilatation in the SB with non-compliant Glider N balloon 2.5 mm x 4 mm at 6 atm pressure
8	LAD	D1	- MV stenting using Resolute Onyx stent 3.0 mm x 18 mm at 12 atm pressure - KBI using semi-compliant LAXA balloon 3.0 mm x 12 mm at 10 atm pressure and semi-compliant LAXA balloon 2.0 mm x 10 mm at 6 atm pressure - Post-dilatation in the MV done twice
9	LAD	D1	- Predilatation in the MV with non-compliant Euphora balloon 2.5 mm x 15 mm at 11 atm pressure - MV stenting with Resolute Onyx stent 3.0 mm x 34 mm at 12 atm pressure - Post-dilatation in the MV - KBI with non-compliant Euphora balloon 3.0 mm x 15 mm at 12 atm in the MV and semi-compliant LAXA balloon 2.5 mm x 15 mm at 12 atm pressure in the SB

### Pre-stenting OCT imaging and 3D reconstruction of full plaque model

Angiography and OCT were performed for all patients prior to stenting. Angiographic images from two projections (separated by > 30°) were used to extract bifurcation centerlines. OCT imaging was conducted using the OPTIS Integrated System (Abbott, Chicago, IL, USA) with an automatic pullback speed of either 18 mm/s or 36 mm/s.

**Fig. 1:**
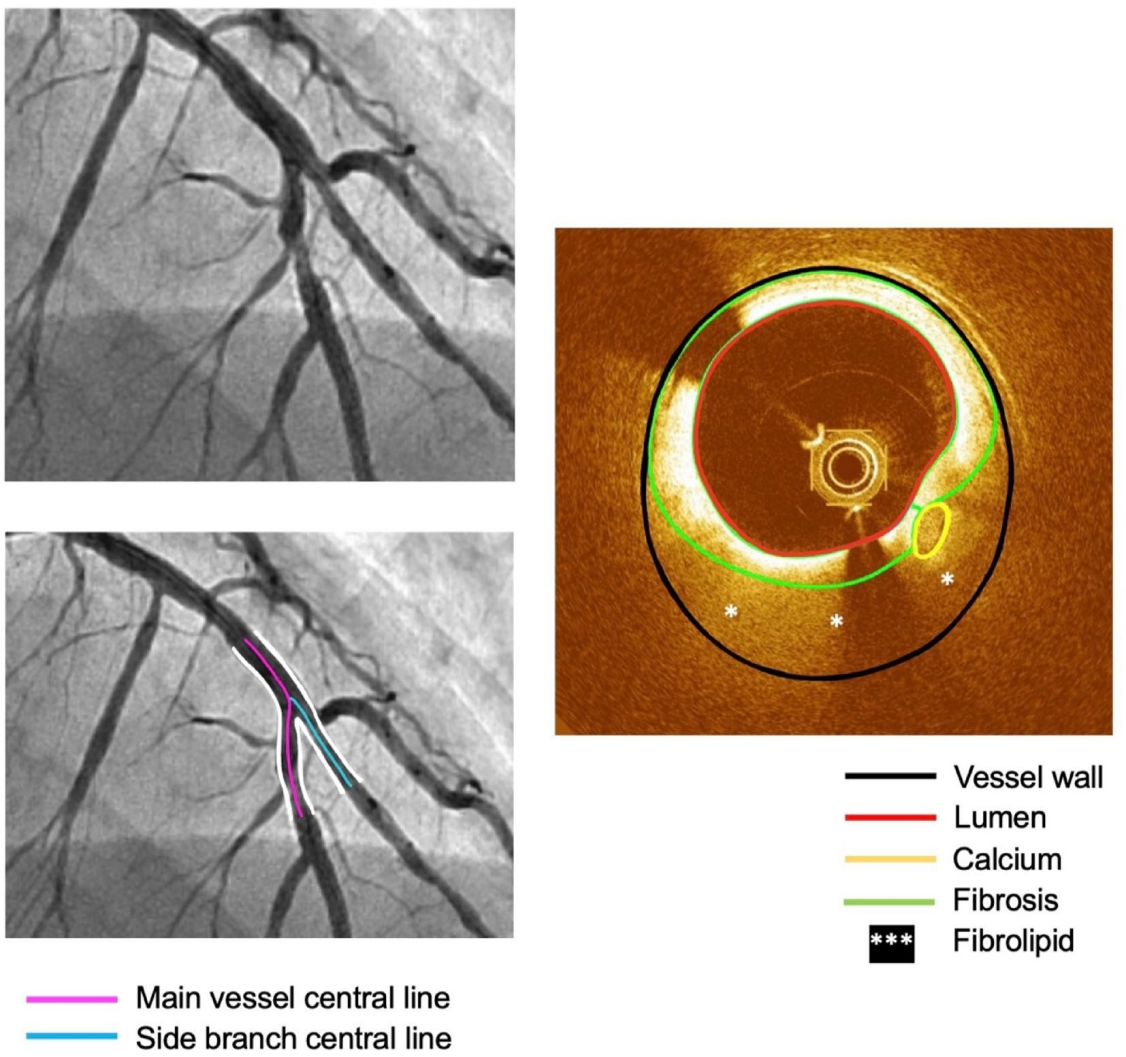
Central lines and OCT segmentation.

**Fig. 2:**
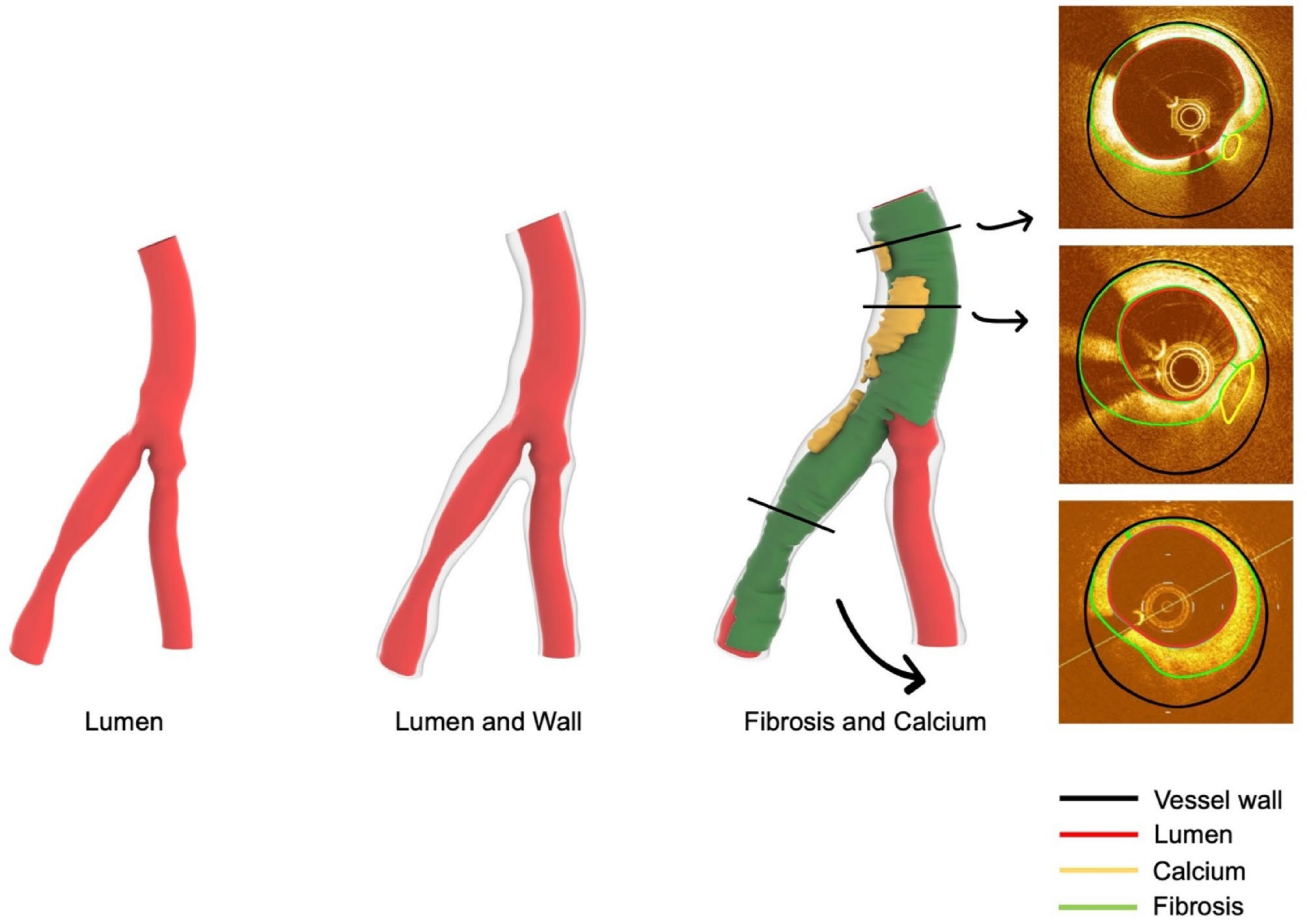
3D reconstruction of the coronary artery from OCT.

OCT images were segmented using echoPlaque 4.0 (INDEC Medical Systems, Los Altos, CA, USA) to delineate the vessel lumen, wall, and plaque components (fibrosis, fibrolipid, and calcium), as shown in Fig. 1. Plaque types were identified based on OCT imaging characteristics: fibrous plaques exhibited homogeneous, highly backscattering regions; fibrolipid plaques presented as areas with diffuse borders, low signal intensity, and significant signal attenuation; calcified plaques appeared as sharply delineated regions with low backscatter and characteristic signal dropout behind the calcification. The 3D coronary bifurcation lumen and wall were reconstructed by fusing the OCT-based segmentation with the extracted bifurcation centerlines to form the basis for the full plaque (FP) model. The plaque geometry was reconstructed strictly following OCT segmentation, and the mapping-back technique was applied to ensure anatomical fidelity^8^ (Fig. 2).

The segmentation and reconstruction process were manual and inherently labor-intensive, but performed by analysts with over 5 years of experience using a standardized protocol to minimize inter-operator variability. All segmentations were independently reviewed by a second operator for consistency. This protocol ensured reproducibility of the 3D reconstructions^8^.

### Material assignment of the full plaque model

**Fig. 3:**
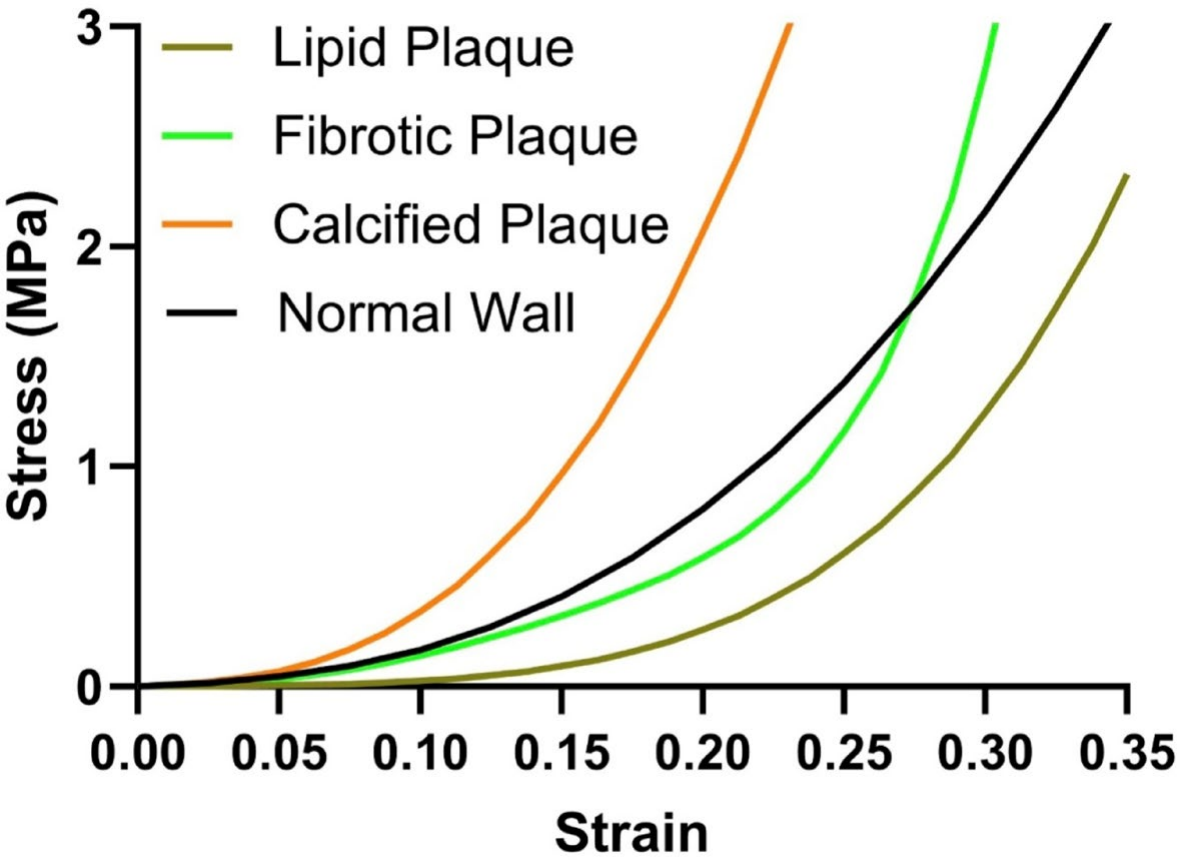
Material properties of the normal wall and plaque materials.

The FP model was meshed with tetrahedral elements. Plaque components were assigned mechanical properties based on literature-derived material data for fibrous, lipid, and calcified plaques^7,9–12^. Vessel walls were modeled as hyperelastic materials, while plaques were modeled as nonlinear elasto-plastic properties^9^ (Table 2**and** Fig. 3).

**Table 2:** Coefficients for the material models assigned for the artery and different plaques.

	$\:{\text{C}}_{10}$(MPa)	$\:{\text{C}}_{20}$ (MPa)	$\:{\text{C}}_{30}$ (MPa)	$\:{\text{C}}_{40}$(MPa)	$\:{\text{C}}_{50}$ (MPa)	C_60_ (MPa)
Normal wall	0.11	3.25	-0.34	0.09	-	-
Calcified plaque	0.11	9.06	-	-	-	-
Fibrotic plaque	0.06	4.28	-21.36	69.36	-	-
Lipid plaque	0.01	0.49	4.13	-	-	-

### Computational stenting simulation

Finite element analysis (FEA) was conducted to simulate patient-specific PCI procedures using the full plaque model, accurately replicating clinical steps such as stent deployment, proximal optimization technique (POT), simultaneous kissing balloon inflation (KBI), and post-dilatation. Simulations were performed using the Abaqus/Explicit solver (Dassault Systèmes, Providence, RI, USA). The stent and balloon were positioned along the lumen centreline, with clinically relevant pressures applied to the balloon’s inner surface. Balloon inflation and deflation durations were fixed at 0.05 s. To approximate quasi-static behavior and minimize inertial effects, mass scaling was applied with a target time increment of 5 × 10⁻⁸ seconds. Both the bifurcation ends and balloon edges were constrained to eliminate rigid body motion. A robust general contact algorithm with a friction coefficient of 0.2 was used to capture interactions between the stent, balloon, and arterial wall. The stress state from each procedural step was propagated to the next to preserve mechanical continuity. Given the high element count and complex contact behavior, simulations were executed on a high-performance computing cluster (2×Intel Sandy Bridge E5-2670, 2.6 GHz, 32 GB 1600 MHz RAM [2 GB/core]) at the University of Miami, with each case run using 128 cores. The predilatation step took approximately 1.5 h, while stenting and post-dilatation required around 5 h, with total simulation time varying based on procedural complexity.

### Post-stenting OCT imaging and lumen segmentation

The post-stenting OCT images were segmented to obtain the post-stenting lumen geometry. The lumen diameters were measured at multiple cross-sectional locations along the stented segment to serve as the ground truth for FEA model validation. The segmentation and analysis of the post-stenting OCT images were conducted using the same methodology as the pre-stenting OCT analysis.

### Statistical analysis

Bland-Altman (BA) analysis was conducted to evaluate the agreement between the simulated models and the post-stenting OCT measurements. The analysis involved calculating the mean differences and the limits of agreement between the simulated and actual post-stenting lumen diameters.

## Results

### Accurate replication of multi-step stenting procedures

**Fig. 4:**
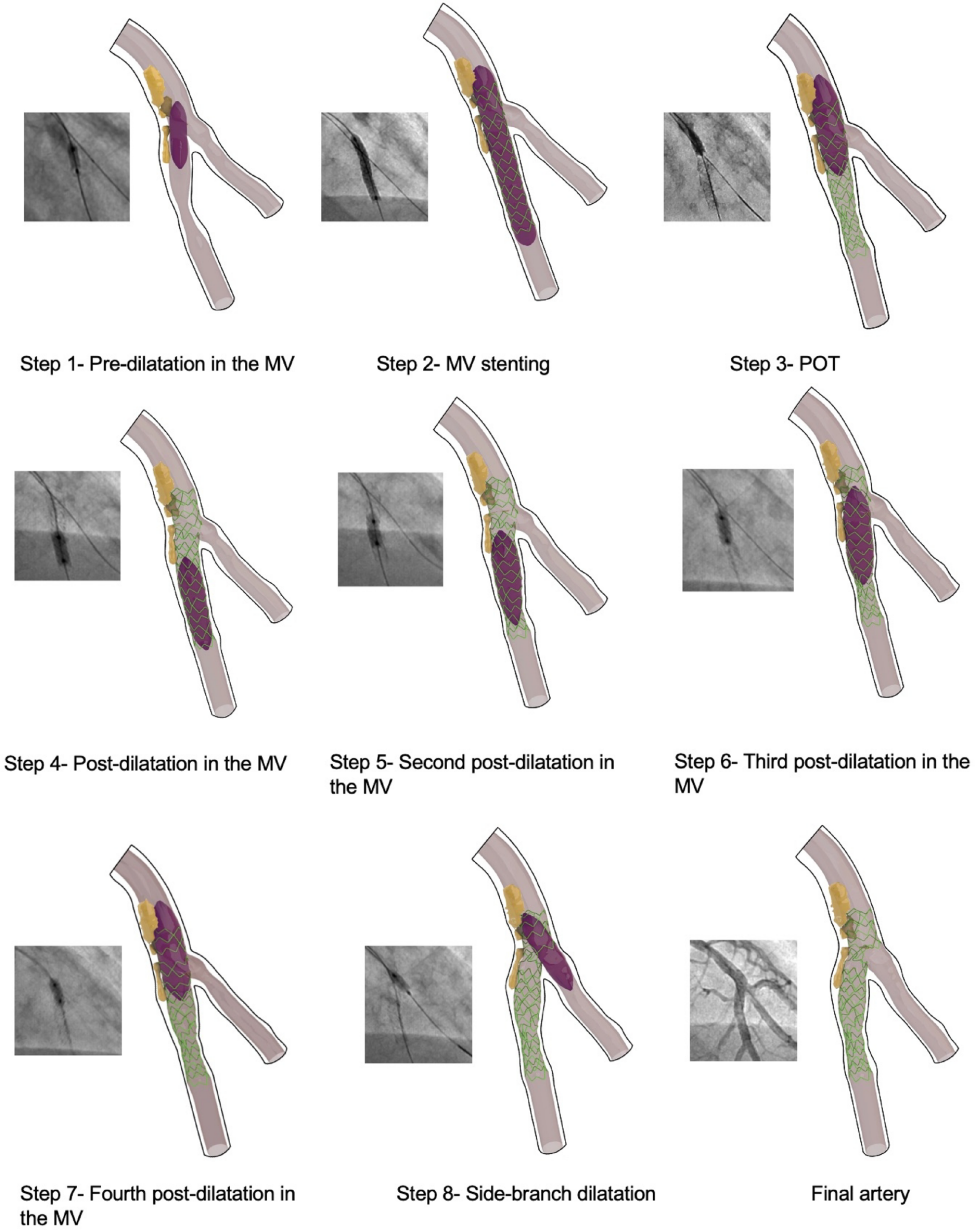
Steps of computational stenting simulation and correlation with real procedural steps.

The FP model successfully replicated each step of the multi-stage stenting procedure, accurately capturing the sequential changes in vessel geometry and stent deformation. Figure 4 illustrates the results for Case 1, comparing simulation outputs to the corresponding angiographic images for each step: predilatation in the main vessel (MV); stent deployment in the MV; POT in the MV; post-dilatation in the MV; and side-branch dilatation.

For improved visualization, only calcified plaques are shown for the simulation results. The FP model effectively captured the deformation of the balloon, stent, and lumen at each stage, aligning closely with the angiographic observations. Notably, the FP model also visualized the sequential deformation of plaques, providing insights into their biomechanical responses during the stenting process.

### Agreement of mean lumen diameter (MLD) with post-stenting OCT

**Fig. 5:**
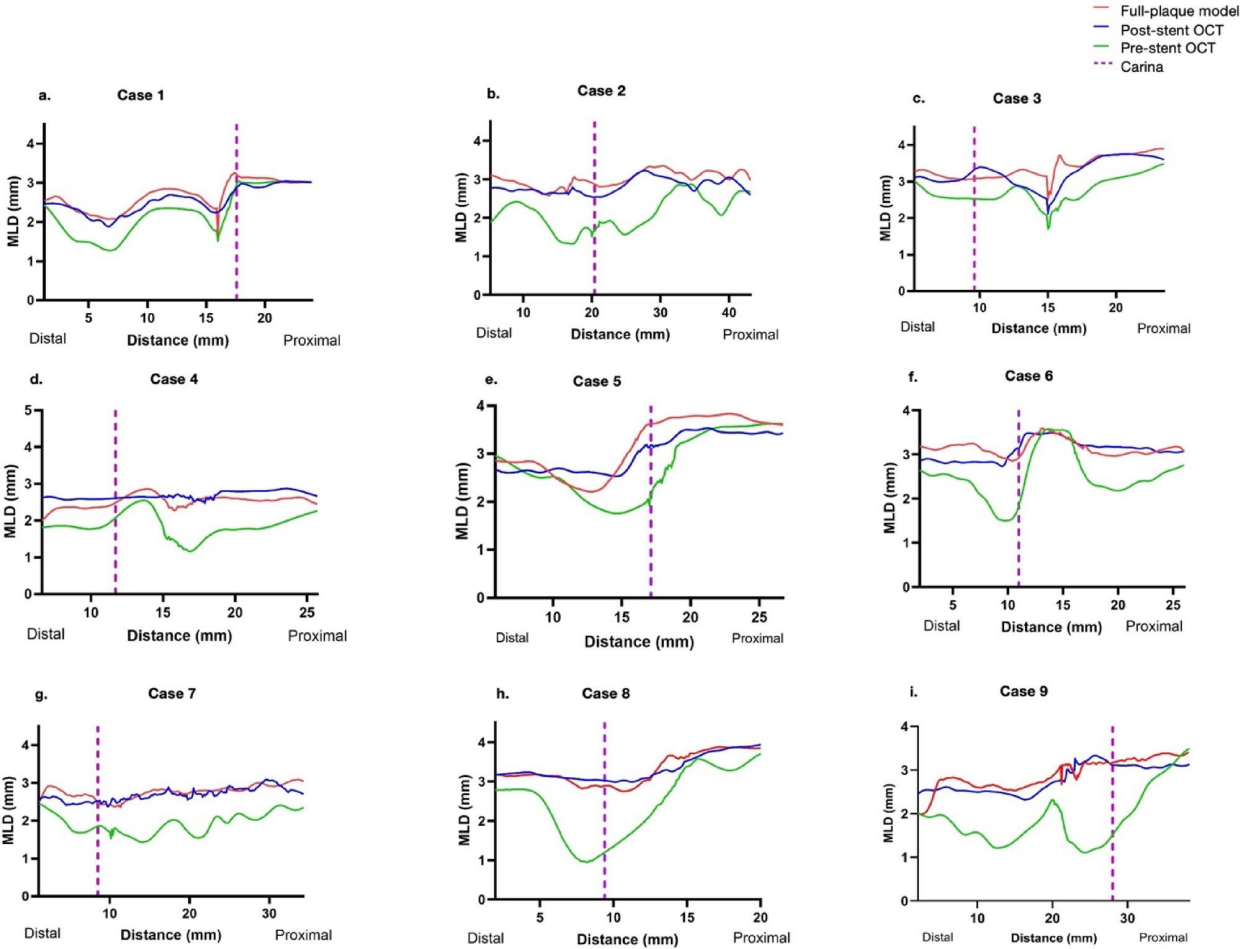
Line plot showing MLD of the full-plaque model, post-stent OCT, and pre-stent OCT.

The FP model’s accuracy in predicting lumen geometry was further validated by comparing the MLD of the simulated model with post-stenting OCT measurements across all nine cases. Figure 5 illustrates the MLD profiles, demonstrating that the FP model closely matched the post-stent OCT data. In contrast, significant differences were noted when comparing pre-stenting OCT data to post-stenting results, underscoring the substantial geometric changes induced by stenting.

### Bland-altman analysis for validation

**Fig. 6:**
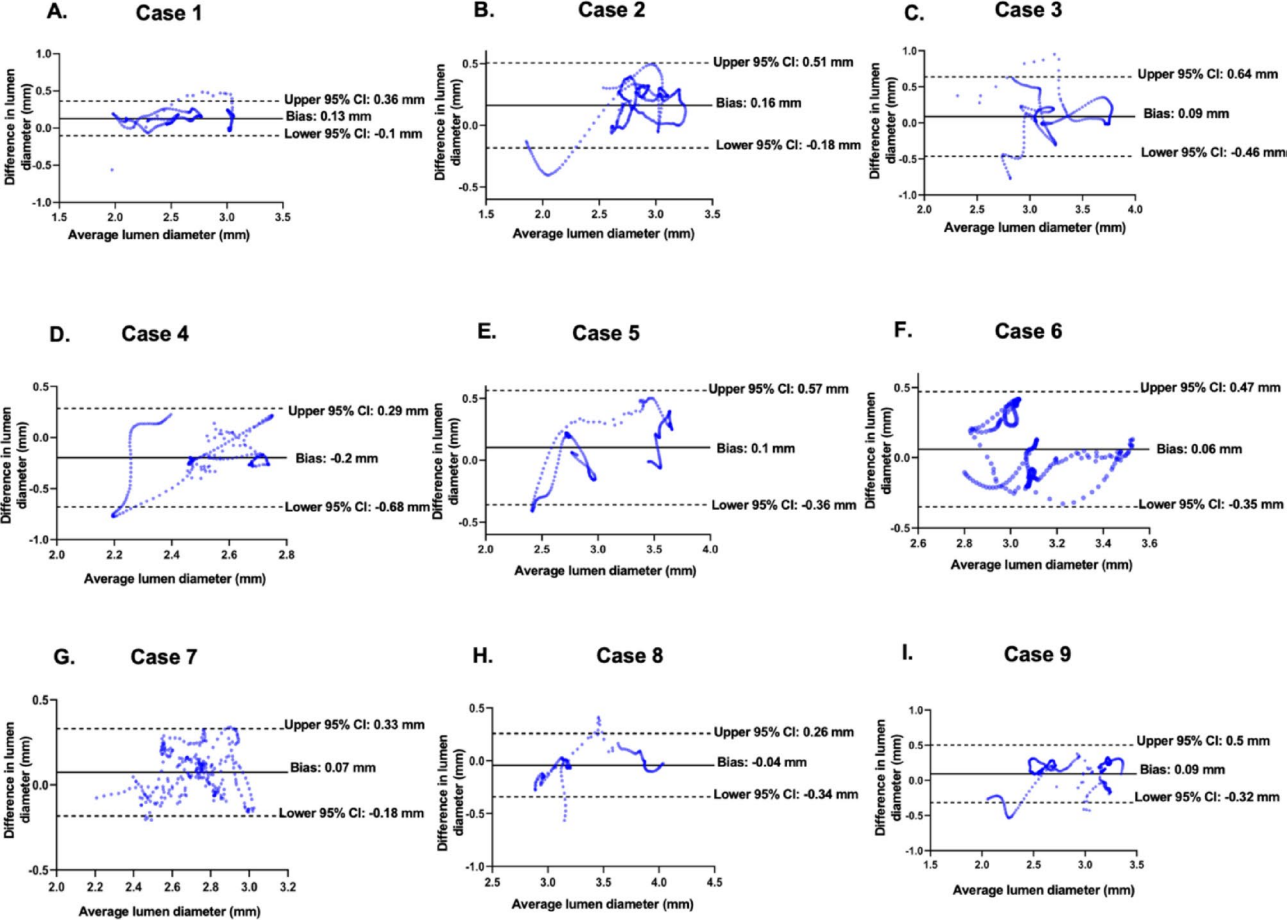
Bland-Altman analysis comparing the MLD measurements between the full-plaque model and post-stent OCT imaging for each patient.

**Fig. 7:**
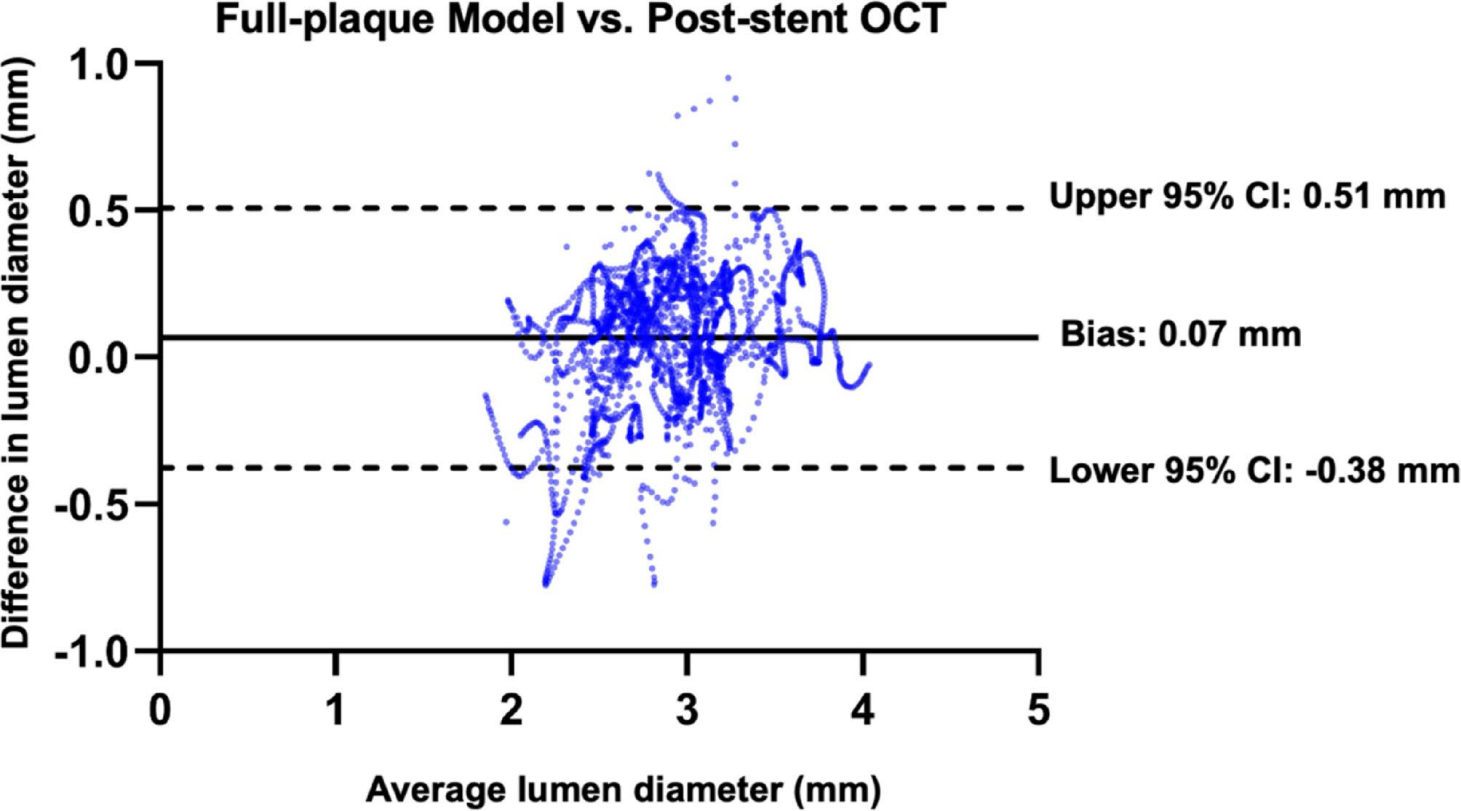
Bland-Altman analysis comparing the MLD measurements between the full-plaque model and post-stent OCT imaging across all cases.

To quantitatively assess the FP model’s accuracy, BA analysis was performed for MLD comparisons between the FP model and post-stenting OCT measurements. The results are presented in Figs. 6 and 7: For each of the nine cases, the FP model demonstrated agreement with post-stenting OCT (Fig. 6; **Supplemental Fig. 1**). Across all cases, the BA analysis revealed a mean bias of 0.07 mm (2.1% error), with 95% limits of agreement ranging from − 0.38 mm to 0.51 mm (Fig. 7; **Supplemental Fig. 2**). These results confirm the FP model’s high accuracy in replicating stent-induced changes in lumen geometry.

### Accurate representation of lumen shape and stent strut distribution

**Fig. 8:**
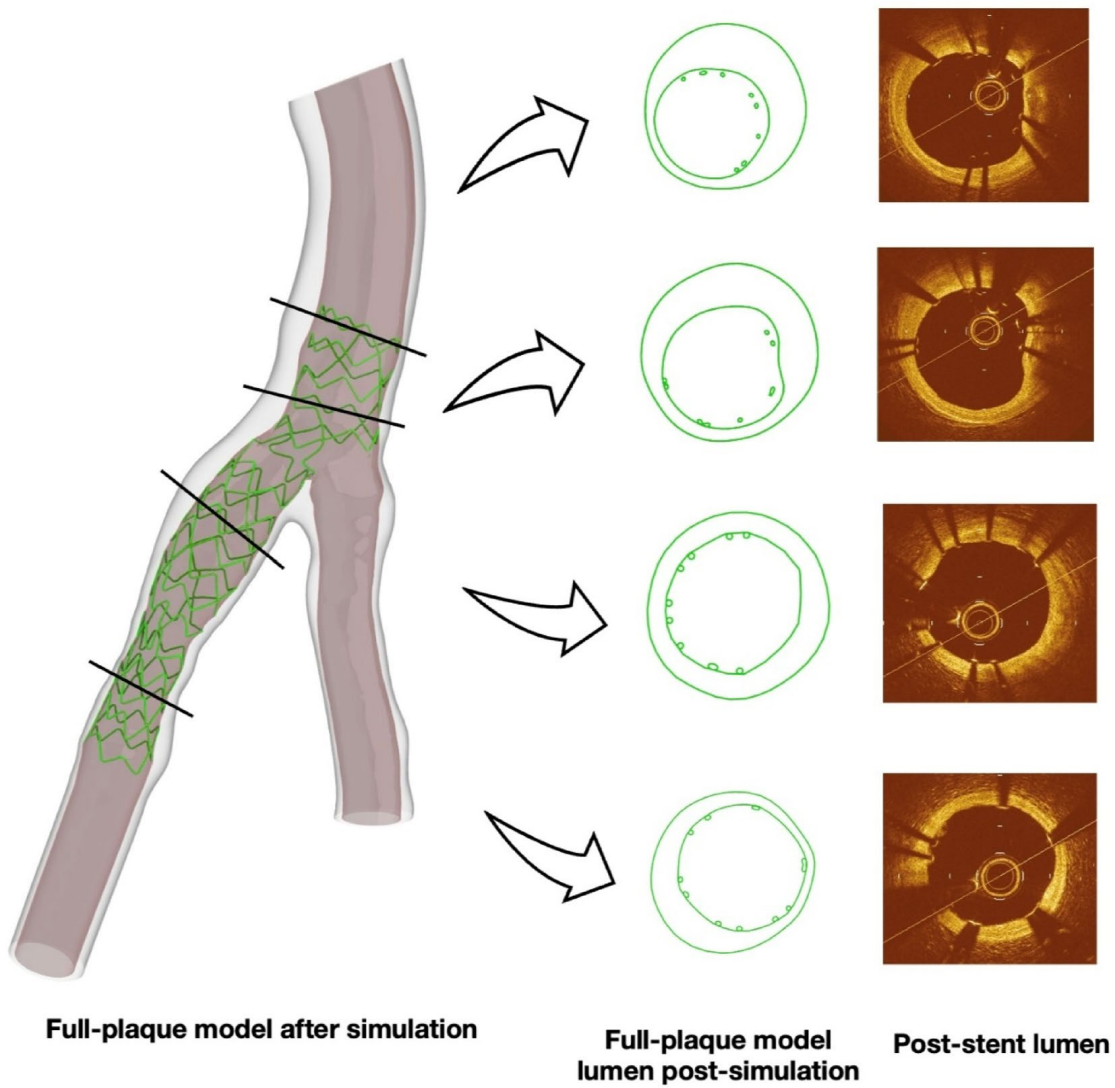
Cross-sectional views of the lumen and stent strut distribution of full-plaque model after simulation and post-stent lumen.

Figure 8 displays cross-sectional views of the post-stenting lumen and stent strut distribution for case 1, comparing the FP model’s predictions with the observed post-stenting OCT data. Despite the complexity of an eight-step stenting procedure, the FP model accurately reproduced the lumen shape and wall deformation. Additionally, the FP model captured the detailed stent strut distribution, including areas of malapposition at the proximal segment. These features are critical for identifying potential mechanical issues, such as areas of high stress or incomplete stent apposition, that could impact long-term outcomes.

## Discussion

The FP model presented in this study is the most detailed and complete simulation of diseased coronary bifurcations developed so far. We used high-resolution OCT imaging, which allowed precise segmentation of the lumen, vessel wall, and different plaque types (fibrosis, fibrolipid, and calcium). This detailed segmentation enabled accurate 3D reconstruction of bifurcation anatomy, capturing complex vessel shapes and plaque compositions^8^. The material properties assigned to plaques and vessel walls were based on established experimental studies and validated computational models^7,9–12^. By incorporating these detailed anatomical and material features, the FP model provides a solid framework for accurately simulating interactions between stents and coronary bifurcations.

Our FP model successfully replicated realistic stenting procedures, which had not been thoroughly achieved previously (Fig. 4). This achievement has important implications for both clinical practice and engineering. First, the model offers valuable insights for personalized clinical strategies. By simulating different stent deployment methods and plaque interactions, clinicians can identify potential issues such as malapposition or incomplete stent expansion. This information can guide decisions about stent selection (e.g., more flexible designs for curved or heavily calcified vessels), balloon materials, and specific procedural strategies, ultimately improving patient-specific care and outcomes, such as reduced restenosis rates. Second, the FP model can be used systematically to evaluate new stent designs aimed at enhancing procedural success. For example, the model can test variations in stent strut thickness, geometry, and flexibility, optimizing designs to suit specific lesion anatomies and plaque characteristics. Third, the FP model can serve as a benchmark for simpler, faster computational models. Simplified models reduce computational demands, but their reliability needs to be validated against more detailed models like the FP model, balancing accuracy and computational efficiency^13^.

Furthermore, given the demonstrated precision in replicating post-stent geometries via high‑resolution OCT, our FP model is also suitable for inverse finite-element applications. Such approaches have been successfully applied to coronary imaging: for example, inverse-FE methods using OCT/IVUS have extracted patient-specific plaque and vessel-wall properties from paired imaging datasets^14^and atherosclerotic plaques have been characterized biomechanically in silico with high accuracy (R² > 0.9)^15^. Together, these findings support the viability of using our validated FP model as a platform for deriving detailed plaque mechanical behavior directly from clinical OCT data.

Regarding discrepancies between simulation results and post-stenting OCT measurements, two main factors could contribute to these variations. One is that OCT imaging artifacts may cause inaccuracies in pre- or post-stenting segmentations, leading to either overestimation or underestimation of lumen dimensions. The other is that errors in simulation might accumulate over multiple sequential stenting steps. Despite these discrepancies, their clinical impact is expected to be small because they are localized and relatively minor. In clinical practice, when clinicians refer to simulation results, they primarily focus on overall vessel geometry, appropriate stent sizing, and comprehensive stent behavior rather than minor, isolated deviations when making treatment decisions.

Despite its strengths, our FP model has several limitations. First, the small sample size (nine patients) limits generalizability. This study was primarily a proof-of-concept to validate technical accuracy. Future studies should include a larger patient group to capture broader anatomical and pathological variations. Second, the model’s development is currently labor-intensive, requiring extensive imaging, manual segmentation, and 3D reconstruction, which affects efficiency. Automation of image segmentation and the introduction of semi-automatic reconstruction techniques are important future goals to streamline the model’s clinical application. Finally, our qualitative assessment of stent strut distribution and malapposition did not include quantitative strut-level comparisons due to challenges like variable visibility of struts, shadowing artifacts, and lack of standardized analysis protocols. Developing standardized quantitative assessment methods is another important direction for future research.

## Conclusion

This study presents a validated full-plaque (FP) finite element model as a detailed and reliable tool for simulating coronary bifurcation stenting. Built upon high-resolution OCT-based reconstruction and literature-supported material properties, the model accurately replicates complex procedural steps and post-stenting vessel geometries. Its strong agreement with post-procedural OCT data confirms its value as a benchmark for assessing simplified simulation methods. Beyond validation, the FP model holds promise for guiding personalized stent planning, optimizing device designs, and supporting inverse modeling to extract patient-specific plaque mechanics. Future efforts will focus on expanding patient data, automating reconstruction processes, and enabling real-time clinical application.

## Supplementary Information

Below is the link to the electronic supplementary material.

Supplementary Material 1

## Data Availability

The OCT data used in this study have been uploaded to the Open Science Framework (OSF) database and are publicly accessible at DOI [10.17605/OSF.IO/5B9XU](https:/osf.io/5b9xu). Any additional data not included in the database is available from the corresponding author upon reasonable request.
