# Supplementary material for: Computational simulations of coronary artery bifurcation stenting using realistic plaque distribution and materials
Source: Sci Rep. 2025 Oct 1;15:34242. doi: 10.1038/s41598-025-16258-0 (PMC12489092; doi:10.1038/s41598-025-16258-0)
Supplement: Supplementary file 1 — Supplementary Material 1 [file 41598_2025_16258_MOESM1_ESM.pdf]

Computational Simulations of Coronary Artery Bifurcation Stenting Using Realistic Plaque Distribution and Materials

Wei Wu, PhD[1]; Shijia Zhao, PhD[1]; Rakshita Ramesh Bhat, M.B.B.S[1]; Yash Vardhan Trivedi, M.B.B.S[1]; Parth Munjal, M.B.B.S[1]; Rahul Chikatimalla, M.B.B.S[1]; Ruben K.A. Tapia-Orihuela, M.D[1]; Hammad Zafar, M.B.B.S[1]; Haritha Darapaneni, M.B.B.S[1]; Komal Arora, M.B.B.S[1]; Changkye Lee, PhD[1]; Yiannis S. Chatzizisis, M.D, PhD[1*]

[1]Center for Digital Cardiovascular Innovations, Cardiovascular Division, Miller School of Medicine, Miami, FL, USA

Corresponding author:
*Yiannis Chatzizisis, MD, PhD
Division of Cardiovascular Medicine
University of Miami Health System
Leonard M. Miller School of Medicine
University of Miami
1120 NW 14th Street, Suite 1124, Miami, FL, 33136
Email: ychatzizisis@icloud.com (YSC)

Supplementary figure legends:
Supplementary Figure 1. Bland-Altman analysis comparing the MLD measurements (percentage) between the full-plaque model and post-stent OCT imaging for each patient

Supplementary Figure 2. Bland-Altman analysis comparing the MLD measurements (percentage) between the full-plaque model and post-stent OCT imaging across all cases

Supplementary figures:

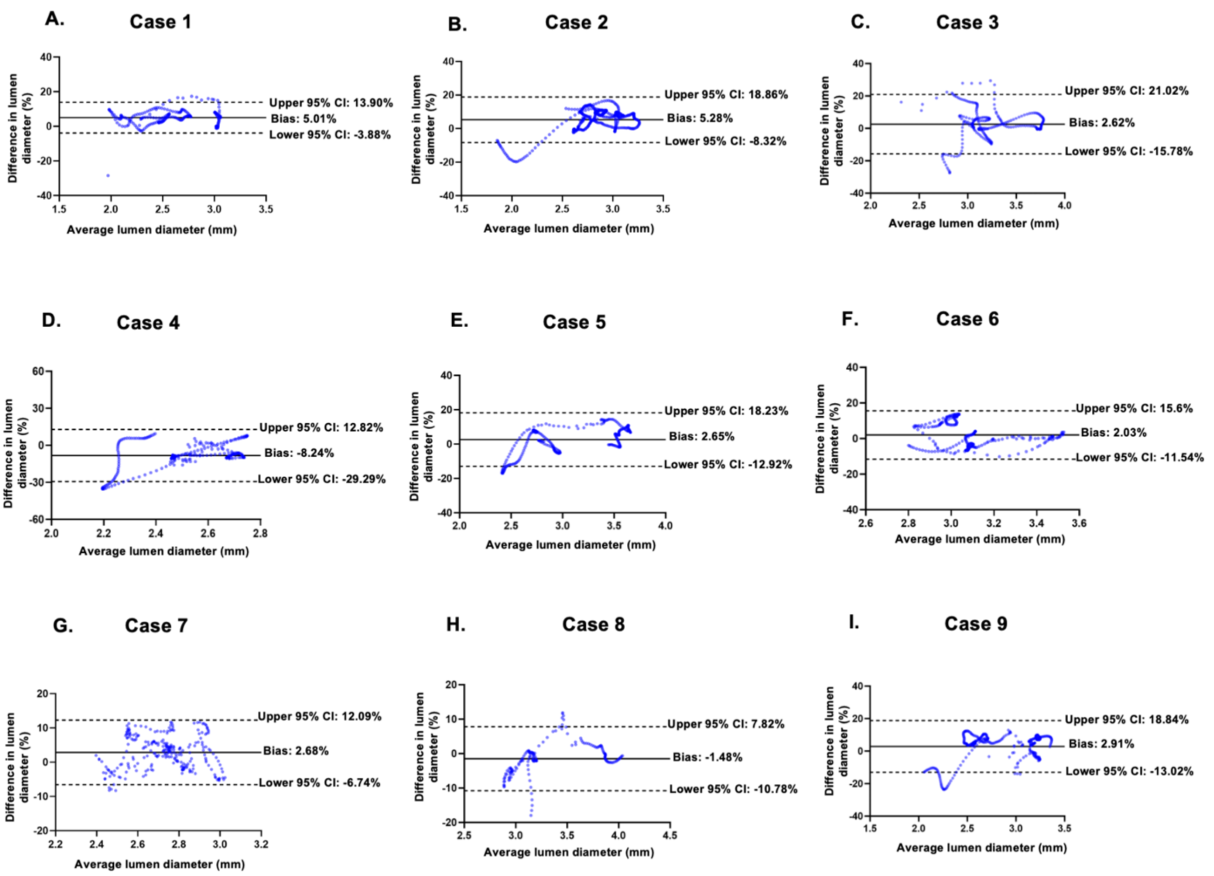

Supplementary Figure 1. Bland Altman analysis showing MLD comparison (percentage) between full-plaque model and post-stent OCT

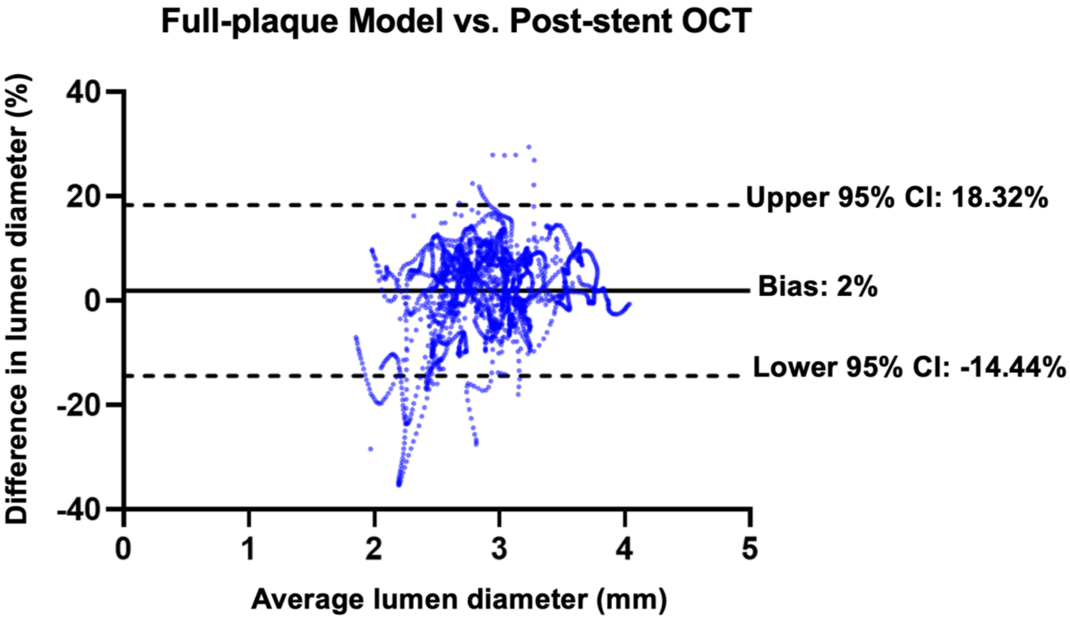

Supplementary Figure 2. Bland Altman analysis showing MLD comparison (percentage) between full-plaque model and post-stent OCT across nine cases
